# Development and psychometric properties of the Knowledge and Attitudes to Mental Health Scales (KAMHS): a psychometric measure of mental health literacy in children and adolescents

**DOI:** 10.1186/s12887-021-02964-x

**Published:** 2021-11-13

**Authors:** Nicola J. Simkiss, Nicola S. Gray, Chris Dunne, Robert J. Snowden

**Affiliations:** 1 Department of Psychology, Swansea University, Swansea, SA2 8PP Wales, UK; 2 Caswell Clinic, Swansea Bay University Health Board, Bridgend, UK; 3 Action for Children, Head Office: 3 The Boulevard, Ascot Road, Watford, UK; 4 School of Psychology, Cardiff University, Cardiff, UK

**Keywords:** Mental health literacy, Mental health, Questionnaire, Psychometric properties, Help-seeking, Self-stigma, Knowledge, Social desirability, Avoidant coping, Stigma

## Abstract

**Background:**

Adolescence is a crucial period for the acquisition of good mental health behaviours, which are the foundation for health and wellbeing in later life. Improved knowledge about mental health and improved help-seeking behaviours have been shown to lead to better mental health outcomes. Mental health literacy (MHL) is multifaceted (e.g., knowledge about symptoms, the stigma around mental health, good mental health practices, etc.). Measures are needed that can assess these different aspects of MHL. Measurement of mental health literacy is currently limited due to a lack of reported psychometric instruments with known psychometric properties. Given that most mental health problems start in early adolescence, a scale is needed that is reliable and valid in this age group.

**Methods:**

The development and validation of the psychometric instrument (termed the Knowledge and Attitudes to Mental Health Scales: KAMHS) entailed two phases: 1) item generation based on an evidence-based intervention programme: The Guide; and 2) item reduction through exploratory factor analysis (EFA), and confirmatory factor analysis (CFA) for factor structure and psychometric assessment. Participants were 559 Year 9 pupils in secondary schools across Wales aged between 13 and 14 years.

**Results:**

Results from the CFA indicated an acceptable fit of the model to the data. The KAMHS showed good internal constancy and moderate test-retest validity (.40–.64).

**Conclusions:**

The final version of the KAMHS contains 50 items that are appropriate for use in children and adolescents. These results suggest that the KAMHS can be used over time to assess the efficacy of interventions aimed at increasing the mental health literacy of adolescent populations.

**Supplementary Information:**

The online version contains supplementary material available at 10.1186/s12887-021-02964-x.

## Background

Children and adolescents’ mental health problems appear to be rising, with an increase in the prevalence by 15% between 1999 to 2017 in the UK, resulting in 1 in 8 children aged 5 to 19 having a mental disorder [1]. In particular, emotional disorders appear to be undergoing a sharp rise, with an increase of around 35% occurring between 1999 to 2017 in 5–15-year olds [1]. High rates of mental health problems are also apparent in other countries [2]. Despite these increases, few of these children appear to be receiving the support or intervention they need [3]. The reasons for this lack of treatment are complex. However, it seems likely that a lack of knowledge and ignorance about mental health problems, and stigma related to mental health difficulties, may contribute to this situation.

### Mental health literacy

Research has often shown that improved knowledge about mental health and mental disorders, and better help-seeking behaviours, have improved mental health outcomes [4]. Mental health literacy (MHL) refers to knowledge and attitudes about mental disorders that help individuals recognise, manage, and prevent mental health disorders [5]. MHL has been shown to be significantly associated with mental health [6, 7]. Inadequate MHL levels have been found to be associated with moderate to severe levels of depression in adolescents [8]. More recently, research has demonstrated that positive MHL significantly associated with improved mental wellbeing in adolescents in Norway [7].

Currently, there are low levels of MHL in secondary schools [9]. Poor MHL is a crucial barrier to help-seeking behaviour [10]. Sufficient knowledge of mental illness and mental health treatments are known to be positively associated with help-seeking and disclosure [4]. Based on a representative survey of English adults, Rüsch et al. identified that better knowledge and understanding of mental illness and positive attitudes were associated with stronger intentions to seek help and disclose mental health problems to family or friends [4].

MHL is multifaceted. At one level, which we term “knowledge”, MHL is an understanding of mental health problems that include information about symptoms of various mental health disorders, the causes of mental health problems, treatment for mental health problems, etc. Definitions also tend to include information about behaviours that maintain or promote “good mental health” [11]. Second, some measures also include the assessment of stigma associated with mental health problems. However, “stigma” is also multifaceted. “Personal stigma” is a person’s view that people with mental health problems are inferior in some manner (with “public stigma” being the amalgamation across a population of these personal stigmas, and “perceived public stigma” being what an individual perceives as the public’s view – which may then influence their own “personal stigma”). “Self-stigma” is what a person who has a mental health problem (or might develop a mental health problem) thinks, or would think, about him/herself in relation to the mental health problem and any associated negative beliefs or attitudes (e.g. “I am weak”). Such self-stigma may result from the internalisation of their “perceived public stigma”.

There is now plenty of evidence that stigma about mental health problems is a significant contributor to people failing to report mental health problems and failing to seek or maintain treatment for their problems both for adults [12] and for adolescents [13]. Research has often shown high mental health stigma and low mental health knowledge about depression and schizophrenia [14, 15].

Interestingly, it appears that different forms of stigma may be associated to that of different forms of the lack of help-seeking behaviours. Research has shown that self-stigma and perceived public stigma had a differential impact upon attitudes towards formal and informal types of help-seeking [16]. They showed that people with higher self-stigma levels had negative attitudes towards formal help-seeking compared to non-formal help-seeking, such as from other college students. In order to reduce the stigma attached to mental illness, education programmes to improve MHL and promote contact with persons with mental illness have often been suggested [17]. Examining stigma as a component of MHL is useful as such negative attitudes can obtain distortions of knowledge and understanding of mental health [18].

### Review of mental health literacy measures

While there are several MHL measures [19, 20], there are several reasons for developing a new measure. This includes several methodological limitations surrounding current measures of MHL. A recent review identified a lack of psychometrically sound MHL scales for use in children, highlighting a significant limitation in the current methods of child-focused MHL [21]. Several MHL reviews have also been conducted [22, 23], which have evidenced low reporting of psychometric properties for these scales. A recent review found that only 38% of MHL knowledge-based measures reported psychometric properties [24]. This makes it difficult to determine a measure’s value and whether it has robust psychometric properties.

There are also conceptual differences in the definition provided for MHL over the years. The original definition of MHL provided by Jorm et al. [11] is illness-oriented [25]. This approach neglects a measure of health-oriented literacy that covers help-seeking behaviours and the importance of good mental health behaviours and positive mental health [26]. As a result, research has provided a broader definition of MHL, which includes knowledge of maintaining good mental health and self-efficacy, rather than a focus on illness [27]. These researchers [27] conceptualise MHL to include four domains: 1) understanding how to obtain and maintain good mental health; 2) understanding mental disorders and their treatments; 3) decreasing stigma against mental illness, and 4) enhancing help-seeking efficacy. Inclusion of all these elements is missing from current measures of MHL as the most commonly used measure to assess MHL is the Vignette Interview [11] which looks explicitly at vignettes of depression and schizophrenia. This type of MHL measure is limited to knowledge of specific mental disorders, and MHL can ultimately vary by the kind of mental health problem being measured [28].

This definition is consistent with the current construct of health literacy defined by the World Health Organisation [29] who defines health literacy is defined as the ability to understand relevant health information, developing skills to manage risk and understanding and recognising when help is needed.

Another issue is that measures of mental health stigma and attitudes towards mental health may not be answered honestly as individuals may seek to conceal their stigmatic beliefs [30], and many current measures of MHL do not account for this. A meta-analysis found weak correlations between implicit and explicit stigma [31], suggesting that stigma is a complex construct that requires careful examination. This raises concerns about whether people will honestly answer questions about mental health attitudes, behaviour, and stigmatic beliefs. Social desirability is the tendency for people’s responses to conform to cultural expectations, rather than reflecting their true beliefs about a topic or accurately reporting about their past or current behaviour [32]. With research assessing stigma and mental health, participants may respond proffering an overly positive image to others about oneself [33]. Although anonymity is an excellent way to reduce this need for social desirability, a measure of social desirability bias may help identify those people who are reporting such an overly positive image [32] and allows measurement of reporting accuracy.

### Aims for developing a new measure of mental health literacy

Our aim was to develop a reliable multifaceted self-report questionnaire, the Knowledge and Attitudes to Mental Health Scales (KAMHS). We aim to go beyond current measures of MHL and measure knowledge about mental health, good mental health-promoting behaviours, stigma to others, and self-stigma. A review of the literature also suggested that poor coping strategies, such as avoidant coping – see [34], were a barrier to help-seeking behaviours, and so the KAMHS also includes a scale to measure these avoidant coping behaviours. The KAMHS also includes a scale that directly measures help-seeking intentions. Finally, the KAMHS consists of a scale for measuring socially desirable responding outside the domain of mental health issues. A number of items (interspersed throughout the questionnaire) that form a Social Desirability scale that is outside the domain of mental health issues (and not part of the total KAHMS score) that can be used to gauge levels of impression management.

Our initial target population was secondary school students (age 11–16) in UK schools. We aimed to test the KAMHS in pupils aged 13–14 years in Welsh secondary schools as this was needed as an outcome measure for a randomised control trial of a MHL intervention, The Guide Cymru [35]. The Guide Cymru is based upon The Guide, which has shown efficacy in improving MHL in a North American sample [36]. We report the development, scoring, and psychometric properties of the KAMHS.

### Initial development of KAMHS

Individual questions were initially written and derived from the content of the evidence-based MHL intervention: The Guide [37]. The Guide is an evidence-based intervention and resource to improve knowledge and attitudes towards mental health in adolescent populations [36]. The Guide Cymru compromises of six modules which include: 1) mental health and mental illness; 2) mental health stigma myths and realities; 3) knowledge on specific mental illnesses; 4) help-seeking and finding support, and 5) experiences of mental illness; and 6) the importance of positive mental health. Therefore, once a draft of the KAMHS was developed, it was submitted for review by education collaborators. The purpose of this review was to receive feedback on the interpretation of the questions, structure, and readability of the questionnaire. Each member of the group commented on the general layout and appearance of the questionnaire. Following this, two age-appropriate children reviewed the questions to help understand the clarity and accuracy of wording; they also provided feedback on the content and relevance of each question. Following review, the questionnaire was revised, and changes such as question re-wording and phrases were made and formed on the feedback received.

Two early revisions of the KAMHS were developed. See Supplementary materials. A sample of 635 year 9 pupils completed Version 1. Verbal feedback was received from both pupils and teachers that the questionnaire was too long, and students struggled to complete all the items. In light of this feedback, items were reviewed on their performance on an Exploratory Factor Analysis (EFA) and by examining the psychometric properties of the items for possible ceiling/floor effects. Items were deleted/added/rewritten with this in mind. A second sample of 574 year 9 pupils then completed KAMHS Version 2. Items such as avoidant coping were added based on their loading within the factor analysis to produce the final version of KAMHS (version 3). It is this final version of the KAMHS that is the focus of the current paper.

## Method

### Participants

The KAMHS was administered to 559 Year 9 secondary school pupils recruited from four schools in Wales, UK. Three schools were located in Denbighshire, and one in Gwynedd. Participants were aged between 13 and 14 years (268 Females, 282 Males, 9 Prefer not to say). Participants’ sociodemographic description was limited for reasons of anonymity.

### Procedure

All participants completed the KAMHS during morning registration. Data collection was performed in the classroom via pencil and paper questionnaires. Approximately 12 weeks after the initial data collection, the KAMHS was administered again by the same schools (*n* = 329) to evaluate test-retest reliability (150 males, 179 females).

### Measures

KAMHS consisted of 50 items that aimed to measure attitudes to mental health across seven domains: Knowledge (12 items), Good Mental Health Behaviours (6 items), Stigma/attitudes (6 items), (lack of) Self-stigma (6 items), (lack of) Avoidant Coping (5 items), Help-seeking behaviours (7 items) and Social Desirability (8 items), see Additional file 2. Participants were asked to rate their agreement with statements on a five-point Likert scale (Strongly Agree, Agree, Don’t Know, Disagree, Strongly Disagree). The response received a score of 4 if it was “correct” (defined by the authors as responses that are factually correct or show the least stigma, etc.). A score of 3 was assigned if they missed by one category (e.g. responding Agree to a Strongly Agree), and 2 if they were two categories out, etc. Thus, a “Don’t Know” would always score 2 points, while responding Strongly Agree to a question where the “correct” answer was Strongly Disagree got a score of 0. The items were scored so that high scores represented positive attitudes or behaviour (e.g. *a lack of* self-stigma, rather than self-stigma). It was decided that “Don’t Know” would be placed in the middle of the scale as research has often found that respondents are more likely to choose ‘undecided or don’t know’ categories when they are presented off to the side of the scale [38]. Many items are reverse scored, see Additional file 2. Average scores for each subscale are computed and prorated for missing items along with a total MHL score. Total MHL scores are calculated by summing each of the average subscales of Knowledge, Good Mental Health Behaviours, Stigma/attitudes, (lack of) Self-stigma, (lack of) Avoidant Coping, Help-seeking behaviours.

### Data analysis strategy

To examine the empirical structure of the KAMHS, an EFA was conducted on half the sample, followed by a CFA on the remaining sample to cross-validate the EFA structure on Time 1 data. The full sample of 559 pupils was randomly split into two samples using random sample of cases command in SPSS, with 279 in the EFA sample and 280 in the CFA sample [39].

## Results

### Preliminary analysis

Examination of skew and kurtosis indicated that all variables met normality assumptions and were appropriate for EFA and CFA.

#### Sample 1

##### Exploratory factor analysis

Exploratory factor analysis was used to investigate the factor structure of the KAMHS by analysing the relationships between items using half the sample (*n* = 279). Knowledge scale items were not included in the EFA because the scale is more a test of knowledge rather than a set of items that measure a particular attitude. The rest of the data from the KAMHS were subjected to a factor analysis using Principal Component Analysis with Oblimin for oblique rotation due to the assumption that many of the MHL factors in the analysis will be correlated [40]. The Kaiser-Meyer-Olkin was 0.81, indicating the data were sufficient for exploratory factor analysis. Barlett’s test of Sphericity was also significant, (*χ*^2^ (435) = 2718.53, *p* < .001), indicating that there was an adequate correlation between the variables and therefore that EFA was appropriate.

Four factors explained a cumulative variance of 43.21%. The scree-plot also revealed a four-factor solution. Items loading at .40 or greater were inspected to understand these factors. Factor 1 contained all 13 items from the Help-seeking and (lack of) Self-Stigma scale. Factor 2 contained all six items for Good Mental Health Behaviours; Factor 3 contained six items from the (lack of) Stigma scale. The fourth factor contained three items from the (lack of) Avoidant Coping scale (see Table 1).

**Table 1:** Exploratory Factor loadings for the 30 items in the KAMHS (excluding Knowledge-based items)

Item text	***Factor 1***	***Factor 2***	***Factor 3***	***Factor 4***
**Factor 1: Help-seeking & Self stigma**				
If I had a mental disorder, I would not feel ashamed	.69			
If I had a mental disorder, I would not avoid socialising	.41			
I would feel a failure if I had a mental disorder	.73			
If I had a mental disorder, I would feel worthless like I had failed my family	.68			
I would feel weak if I had a mental disorder	.73			
If I had a mental disorder, I would feel I’d let everyone down	.73			
I wouldn’t tell anyone if I had a mental health problem in case they made fun of me	.64			
I am confident that I could ask for help if I had a mental health problem	.59			
For me, it would be easy to ask for help for a mental health problem	.67			
If I had a mental health problem, I would try to hide it from everyone	.71			
If I had a mental health problem, I would be happy to tell my teacher or school counsellor .63				
It’s best not to tell anyone about your mental health problems	.52			
If I had a mental health problem, I would not tell friends and family	.66			
**Factor 2: Good Mental Health Behaviours**				
The same things that help our physical health also help our mental health		.70		
Sometimes things that stress you should be faced head-on		.56		
Healthy eating helps you maintain good mental health		.67		
A good night’s sleep is good for your mental health		.59		
Regular exercise has no effect on your mental health		.45		
Talking about your feelings can help with mental health problems		.43		
**Factor 3: Stigma**				
If my friend had a mental disorder, I would avoid them			.53	
I would not like to be in the same classroom as someone with a mental disorder			.63	
I would feel comfortable sitting next to a person with a mental disorder			.67	
I wouldn’t want to marry or date a person with a mental disorder			.66	
I would be happy for a person with a mental disorder to come to my house			.59	
Mental disorders are caused by people being wicked or bad			.50	
**Factor 5: Avoidant Coping**				
Drinking alcohol never helps when you are stressed^a^				
It’s often best to ignore problems and hope they go away				.42
Taking illegal drugs can never help when you are stressed by something^a^				
The best way to cope with problems is not to think about them				.53
I do my best not to think about my problems				.58

^a^Did not load

##### Confirmatory factor analysis

As the data is ordinal, a confirmatory factor analysis using a diagonally weighted least squares (DWLS) estimator to determine whether the factor structure obtained using exploratory factor analysis could be confirmed on the second half of the sample. For the CFA, we decided to keep help-seeking behaviours and a lack of self-stigma scales separate as these constructs would be performed separately with future research as self-stigma may have distinct and significant contributions to other aspects of MHL [41].

Therefore, Help-seeking behaviours, lack of Self-stigma, lack of Stigma, Good Mental Health Behaviours, and lack of Avoidant coping subscales were analysed as recommended by Lavaan package version 0.5–22 [42]. Items 21 and 8 were removed for the confirmatory factor analysis as they did not load in the EFA. A confirmatory factor analysis was then conducted on the remaining 280 participants to determine whether the factor structure required modification. Items 37 and 45 were removed as they loaded poorly (below .15).

The model resulted in an excellent fit as the Comparative Fit Index (CFI), and Tucker Lewis Index (TLI) were both above the recommended value of .90 where ***x***^***2***^ ***(df = 289) = 372.15, CFI = .90, TLI = .90, and RMSEA = .032.*** Each item loaded significantly on its respective latent factor and all specified covariances were significantly different from zero (see Table 2).

**Table 2:** Standardised Factor Loadings in the CFA, Standard Errors and Omega reliability of KAMHS subfactors

Factor and items	ω	λ	SE
***Help-seeking behaviours***	.76		
Q5. I wouldn’t tell anyone if I had a mental health problem in case they made fun of me		.52	0.00
Q11. For me, it would be easy to ask for help for a mental health problem		.68	0.17
Q24. If I had a mental health problem I would try to hide it from everyone		.69	0.19
Q42. If I had a mental health problem, I would be happy to tell my teacher or school counsellor		.59	0.19
Q46. If I had a mental health problem, I would not tell friends and family		.57	0.16
Q1. I am confident that I could ask for help if I had a mental health problem		.44	0.17
Q45. It’s best not to tell anyone about your mental health		–	–
***Lack of Self-stigma***	.80		
Q10. If I had a mental disorder I would not feel ashamed		.56	0.00
Q23. If I had a mental disorder I would not avoid socialising		.42	0.13
Q25. I would feel a failure if I had a mental disorder		.75	0.16
Q33. If I had a mental disorder, I would feel worthless like I had failed my family		.74	0.16
Q43. I would feel weak if I had a mental disorder		.66	0.14
Q49. If I had a mental disorder, I would feel I’d let everyone down		.66	0.15
***Lack of Stigma***	.66		
Q2. If my friend had a mental disorder I would avoid them		.43	0.00
Q16. I would not like to be in the same classroom as someone with a mental disorder		.55	0.35
Q27. I wouldn’t want to marry or date a person with a mental disorder		.68	0.35
Q36. I would be happy for a person with a mental disorder to come to my house		.61	0.36
Q17. I would feel comfortable sitting next to a person with a mental disorder		.48	0.34
Q38. Mental disorders are caused by people being wicked or bad		.16	0.24
***Good Mental Health Behaviours***	.61		
Q34. The same things that help our physical health also help our mental health		.25	0.00
Q35. Sometimes things that stress you should be faced head-on		.35	0.60
Q39. A good night’s sleep is good for your mental health		.43	0.81
Q44. Regular exercise has no effect on your mental health		.24	0.62
Q50. Talking about your feelings can help with mental health problems		.64	1.40
Q37. Healthy eating helps you maintain good mental health		–	–
***Avoidant Coping***	.53		
Q6. It’s often best to ignore problems and hope they go away		.88	0.00
Q47. I do my best not to think about my problems		.24	0.13
Q40. The best way to cope with problems is not to think about them		.33	0.16

Note. *λ* Standardized factor loading, *SE* Standardized error of factor loading

#### Distribution of scores

The psychometric properties of the scales are shown in Table 3. The mean scores for each of the scales of the KAMHS was approximately in the middle of the possible range of scores (0–4), with most of the range being used for all of the scales. Hence, there were no signs of floor or ceiling effects. Levels of skew were small for all the scales. Levels of kurtosis were small for all of the scales, with the exception of the Knowledge scale, which showed leptokurtosis.

**Table 3:** Descriptive statistics for the Knowledge and Attitude to Mental Health Scales

Subscale	Mean (SD)	Range	Skew (SE)	Kurtosis (SE)	Test – retest reliability	Means male: female
Knowledge	2.21 (0.30)	0.42–3.50	0.10 (0.10)	3.63 (0.21)	.40**	2.21: 2.21
Good Mental Health	2.53 (0.51)	0.67–4.00	0.14 (0.10)	0.27 (0.21)	.41**	2.59: 2.49
(Lack of) Stigma	2.94 (0.58)	0.67–4.00	− 0.24 (0.10)	0.08 (0.21)	.47**	2.81: 3.06 #
(Lack of) Self-Stigma	2.35 (0.74)	0.00–4.00	−0.40 (0.10)	0.19 (0.21)	.60**	2.46: 2.25 #
(Lack of) Avoidant Coping	2.25 (0.62)	0.00–4.00	−0.19 (0.10)	0.48 (0.21)	.45**	2.24: 2.27
Help-seeking Behaviours	2.33 (0.73)	0.00–4.00	−0.02 (0.10)	0.23 (0.21)	.64**	2.48: 2.19 #
Social Desirability	2.01 (0.62)	.38–3.75	0.09 (0.10)	−0.16 (0.21)	.56**	1.92: 2.12 #

** Correlation is significant (2-tailed) *p* < .01

# Significant differences between means (2-tailed) *p* < .05

#### Internal reliability

Once the CFA model was estimated, functions from the semTools package [43] were used to obtain reliability estimates based on the CFA model object created by Lavaan. Omega reliability was calculated for the internal reliabilities of the KAMHS are shown in Table 2. (Lack of) Self-stigma (lack of) Stigma, and Help-seeking Behaviours exhibited acceptable or good reliability (ω = .66 to .80) and Good Mental Health Behaviours and (lack of) Avoidant Coping, ω demonstrated poor internal reliability (ω = .61 to .53).

As knowledge and social desirability were not included in the CFA model, alpha reliability was calculated. Social desirability scale had acceptable internal reliability (*α* = .69). The Knowledge scale had poor internal reliability (*α* = .36). However, this low internal reliability might be expected as this scale is not a measure of an “attitude” but a series of questions about mental health issues, and pupils may be aware of some aspects of mental health knowledge but not others, resulting in the poor internal consistency of this scale (See Discussion).

#### Test re-test reliability

Test re-test was completed at 12 weeks as this was the period for delivery of a MHL intervention ‘The Guide Cymru’ that the KAMHS was needed as an outcome measure with the hope that any differences during this period would be minimal. The Guide Cymru consists of 6 modules of MHL and therefore, 12 weeks were provided to allow schools time to deliver the modules of MHL to pupils. Twelve weeks after the initial data collection, the same schools completed the KAMHS, (*n* = 329). Pearson’s correlation coefficients are reported as measures of test-retest reliability. The test-retest reliability for each of the scales of the KAMHS was fair to good (as defined by [44], ranging from .40 to .64.

#### Gender differences

Mean scores for each sub-scale of the KAMHS split by gender are illustrated in Table 3. Female students had higher scores on the (lack of) Stigma scales compared to male students, suggesting that they show less stigmatic attitudes to people with mental health problems. However, female students scored lower than males on the (lack of) Self-stigma and Help-seeking scales suggesting that they internalise stigma more, possibly leading to less disclosure and help-seeking behaviour. Female students also showed greater levels of social desirability. No other sub-scales on the KAMHS showed significant gender differences.

## Discussion

This research aimed to establish a reliable instrument for assessing adolescents’ attitudes toward mental health - The Knowledge and Attitude to Mental Health Scales (KAMHS). This psychometric measure goes beyond other research in generating six scales derived from the literature on MHL which quantify: 1) knowledge of mental health; 2) good mental health behaviours; 3) (lack of) stigma; 4) (lack of) self-stigma; 5) (lack of) avoidant coping, and 6) help-seeking behaviours. The questionnaire also includes a measure of social desirability that can identify participants who present with an overly positive view of themselves.

This new MHL measure began with a pool of items and has gone through several stages of development prior to the current version, being developed via factor-analytic methods [45]. The final version of the KAMHS contains 50 items appropriate for use in children and adolescents of secondary school age that can be completed in 15–20 min. The CFA model has an acceptable fit to the model as the goodness of fit fell within the acceptable range. The psychometric properties of the KAMHS were good overall, showing no signs of floor or ceiling effects, fair to good internal consistencies, and fair to good test-retest reliabilities. Although these test-retest reliability coefficients are only fair to good, they are in line with other instruments that aim to measure similar constructs [32].

The exception to this general pattern of good psychometric properties for the KAMHS was the relatively poor reliability of the Knowledge scale, (Lack of) Avoidant Coping and Good Mental Health Behaviours falling below .61. The Avoidant coping scale was a relatively new addition to KAMHS Version 3 and therefore requires a further review of the items included. Both the Knowledge scale and Good Mental Health Behaviour include several different aspects of knowledge or understanding about mental health, relating to knowledge about various mental health disorders and treatments. It should also be noted that the reliability of questionnaire data has been shown to be much lower in children than in adults [46]. A possible explanation for these differences in reliability may relate to the cognitive development of children and adolescents and the changing cognitive capacities of developing young people, compared to adults [47]. Previous research also considers alternative factors that may impact reliability, such as the influence of classmates during questionnaire completion, poor motivation and boredom, all of which can impact data quality and reliability in child samples [47].

The EFA revealed a four-factor model, combining two original factors (*Help-seeking and self-stigma*). This is not unusual as help-seeking behaviours are often predicted by self-stigma [48] identifying that self-stigma predicted attitudes toward seeking professional psychological help and willingness to seek counselling help above any previously identified predictors such as sex, previous counselling experience, self-esteem and tendency to self-disclose. Therefore, these constructs are positively related, and it is not easy to separate out but for further analysis of these constructs they would be performed separately as both self-stigma and help-seeking behaviour may have distinct and significant contributions to other aspects of MHL [41].

## Strengths and limitations

The main limitation of the present study is that it was confined to the original target population of 13–14-year-old adolescents living in Wales, UK. Further data will be needed to show that the KAMHS is appropriate for use in other age groups. Given that many mental health problems can start before the age of 14 [2], it would be useful to be able to measure MHL reliably in younger age groups. Further, there are clear cultural differences in attitudes to mental health [49, 50] and in MHL [51] and therefore the psychometric properties demonstrated in the present sample may not transfer to other countries and cultures. However, given the robust psychometric properties of the KAMHS demonstrated by the current study, it seems likely that the KAMHS will be valuable in being able to reliably measure MHL in different ages and different cultures.

The KAMHS also contains a measure of social desirability. Such a scale may prove useful in future studies in identifying individuals who give overly positive responses and whose responses may not, therefore, be accurate, or to look at the effects of such socially desirable responding on the relationship between other variables. For example, participants with high levels of social desirability might report high levels of good mental health behaviours, and good levels of mental health, due to this high social desirability and thus bias the results to show a relationship between these other variables, due to all scales being falsely inflated. Recent research has identified social desirability to be significantly negatively related to self-report measures of Externalising problems and positively related to Quality of Life in adolescents. Hence, it appears that some of the variance in reporting on mental health issues may be due to impression management [52].

## Conclusion

The KAMHS has been developed to measure six aspects of MHL: Knowledge, Good Mental Health Behaviours, Stigma/attitudes, (lack of) Self-stigma, (lack of) Avoidant Coping, Help-seeking behaviours. In addition, it contains a separate scale to measure Social Desirability. We demonstrate its factor structure and that the scales have good psychometric properties. Therefore, we believe the KAMHS is a reliable instrument to measure MHL and changes in MHL over time due to educational intervention programmes.

## Supplementary Information

**Additional file 1.**

**Additional file 2.** Knowledge and Attitudes to Mental Health Scales (Version 3).

## Data Availability

The datasets used and/or analysed during the current study are available from the first or corresponding author on reasonable request.

## Abbreviations

MHL: Mental Health Literacy
KAMHS: Knowledge and Attitudes to Mental Health Scales
CFA: Confirmatory Factor Analysis
EFA: Exploratory Factor Analysis
UK: United Kingdom
TLI: Tucker Lewis Index
CFI: Comparative Fit Index
RMSEA: Root Mean Square Error of Approximation
