# Supplementary material for: Development and psychometric properties of the Knowledge and Attitudes to Mental Health Scales (KAMHS): a psychometric measure of mental health literacy in children and adolescents
Source: BMC Pediatr. 2021 Nov 13;21:508. doi: 10.1186/s12887-021-02964-x (PMC8590271; doi:10.1186/s12887-021-02964-x)
Supplement: Supplementary file 1 — Additional file 1. [file 12887_2021_2964_MOESM1_ESM.docx]

Supplementary materials

Knowledge and Attitudes to Mental Health Scales (Version 1).

**Understanding how to optimise and maintain good mental health**

1. Spending time interacting with friends and family helps your mental health. (6) T
2. Drinking alcohol does not help when you are stressed (9) T
3. It’s often best to ignore problems and hope they go away (15) F
4. It is best not to tell anyone if you feel you have a problem (26) F
5. Sometimes taking illegal drugs can actually help when you are stressed by something. (28) F
6. A good night’s sleep helps your mental health. (33) T
7. The same things that help our physical health also help our mental health. (35) T
8. Playing sport is bad for your mental health. (40) F
9. Sometimes things that stress you should be faced head-on (43) T
10. Healthy eating helps you maintain good mental health (51) T
11. Talking to someone can help if you are distressed. (53) T
12. Physical exercise has no effect on your mental health (68) F

**Stigma/attitudes**

1. People with a mental disorder are dirty. (5) F
2. I would feel scared if I was around someone with a mental disorder. (8) F
3. If a person with a mental disorder came to my school, I would try to be friends with them. (11) T
4. People with a mental disorder will not be able to play certain sports such as football. (18) F
5. Having a mental disorder is something to be ashamed of. (27) F
6. People with a mental disorder aren’t really ill and should just get on with things. (34) F
7. Receiving help for a mental disorder is a sign of weakness. (36) F
8. I would still be friends with my best friend if they had a mental disorder. (41) T
9. People with mental disorders are dangerous. (44) F
10. Mental disorder cannot happen to me. (48) F
11. Mental disorders are caused by people being wicked or bad. (54) F
12. I believe someone with a mental disorder should be blamed for their problem. (60) F
13. An individual with a mental disorder should not be allowed to join in classroom activities (63) F
14. I would feel comfortable sitting next to a person with a mental disorder. (70) T

**Understanding mental disorders and their treatments**

1. Anorexia only affects women (1) F
2. Lack of pleasure, hopelessness and feeling tired can all be symptoms of a Depression. (2) T
3. Symptoms of Panic Disorder include moving quickly and feeling that people are staring at you. (3) F
4. Emotions are controlled by your heart. (7) F
5. An Anxiety Disorder happens when a person’s brain detects the presence of danger – such as a dog barking. (10) F
6. Medicines should never be used to treat a mental disorder. (13) F
7. Most mental disorders start before the age of 18 (16) T
8. People with Schizophrenia have a split personality. (19) F
9. Hallucinations are where people hear or see things that aren’t really there. (20) T
10. People who wash their hands have Obsessive-Compulsive disorder (OCD). (23) F
11. Symptoms of Panic Disorder include the heart beating fast, sweating, and feeling dizzy. (25) T
12. Severe and repeated stress can affect the brain. (29) T
13. The most common mental disorders in teenage girls are eating disorders. (30) F
14. Depression is not a true mental disorder (31) F
15. If you are worried about something then you probably have Generalised Anxiety Disorder. (32) F
16. People with Bipolar Disorder have trouble making their minds up. (38) F
17. Anorexia Nervosa is an eating disorder that can lead to death. (39) T
18. People with Bipolar Disorder have periods of clinical Depression and periods of Mania. (45) T
19. Vitamins and yoga are effective treatments for most mental disorders. (47) F
20. Attention Deficits Hyperactivity Disorder (ADHD) is caused by watching too much TV or eating too much sugar. (49) F
21. A delusion is seeing something that is not real.(50) F
22. Everybody feels stressed some of the time. (55) T
23. People on a diet probably have Anorexia Nervosa.(56) F
24. Getting people to talk about a traumatic event immediately after the event helps prevent PTSD. (58) F
25. Three symptoms of clinical Depression are feeling sad, eating a lot, and hearing voices. (61) F
26. Obsessions are thoughts that occur often that the person wants to get rid of but can’t. (62) T
27. Schizophrenia can be treated with medication and psychological therapy. (66) T
28. Depression is usually caused by an event such as falling out with your friends or with your family. (71) F

**Help seeking behaviours**

1. For me, it would be easy to ask for help for a mental health problem. (17) T
2. I wouldn’t tell anyone if I had a mental health problem in case they made fun of me. (21) F
3. If I have a mental health problem, I know I will ask for help. (42) T
4. Asking for help with a mental health problem will probably make it worse (52) F
5. I am confident that I could ask for help if I had a mental health problem. (59) T
6. If one of my close friends needed help with a mental health problem, I would encourage them to seek help. (64) T
7. If one of my family members had a mental health problem, I would encourage them to seek help. (67) T
8. If I had a mental health problem would try to hide it from everyone. (72) F

**Social Desirability**

1. I always eat a healthy diet (4) F
2. I sometimes gossip about others (12) T
3. I am always patient when waiting in a queue (14) F
4. I always do what my parent ask first time (22) F
5. I have pretended to be ill in order to not go to school. (24) T
6. I am always honest. (37) F
7. I always admit when I am wrong (46) F
8. I sometimes think bad thoughts about people (57) T
9. I always keep my promises (65) F
10. I have never dropped rubbish (69) T

Knowledge and Attitudes to Mental Health Scales (Version 2).

**Understanding how to optimise and maintain good mental health**

1. Drinking alcohol does not help when you are stressed (1) (SA)
2. It’s often best to ignore problems and hope they go away (6) (SD)
3. Taking illegal drugs can never help when you are stressed by something (21) (SA)
4. The same things that help our physical health also help our mental health (34) (SA)
5. Healthy eating helps you maintain good mental health (37) (SA)
6. Sometimes things that stress you should be faced head-on (35) (SA)

**Public Stigma (lack of)**

1. People with a mental disorder can be unpredictable at times (2) (SD)
2. I would be happy for a person with a mental disorder to come to my house (36) (SA)
3. Mental disorders are caused by people being wicked or bad (38) (SD)
4. A child with a mental disorder should be carefully watched during classroom activities (16) (SD)
5. I wouldn’t want to marry or date a person with a mental disorder (27) (SD)
6. I would feel comfortable sitting next to a person with a mental disorder (17) (SA)

**Understanding mental disorders and their treatments (Knowledge)**

1. Emotions are controlled by your heart (28) (SD)
2. An Anxiety Disorder happens when a person’s brain detects the presence of danger- such as a dog barking (3) (SD)
3. Medicines should never be used to treat a mental disorder (15) (SD)
4. People with Schizophrenia have a split personality (18) (SD)
5. Most mental disorders start before the age of 18 (7) (SA)
6. Severe and repeated stress can affect the brain (29) (SA)
7. If you are worried about something, then you probably have Generalised Anxiety Disorder (13) (SD)
8. People with Bipolar Disorder have periods of clinical depression and periods of Mania (31) (SA)
9. Vitamins and yoga are effective treatments for most mental disorders (26) (SD)
10. Attention Deficit Hyperactivity Disorder (ADHD) is caused by watching too much TV or eating too much (20) (SD)
11. Obsessions are thoughts that occur often that the person wants to get rid of but can’t (12) (SA)
12. Schizophrenia can be treated with medication and psychological therapy (32) (SA)

**Help-seeking behaviours**

1. For me, it would be easy to ask for help for a mental health problem (11) (SA)
2. I wouldn’t tell anyone if I had a mental health problem in case they made fun of me. (5) (SD)
3. I am confident that I could ask for help if I had a mental health problem (8) (SA)
4. If I had a mental health problem I would try to hide it from everyone (24) (SD)

**Social Desirability**

1. I sometimes gossip about others. (4) (SA)
2. I always do what my parents ask first time (30) (SA)
3. I have pretended to be ill in order to not go to school (22) (SA)
4. I always admit when I am wrong (19) (SA)
5. I have never dropped rubbish (9) (SA)
6. I always keep my promises (14) (SA)

**Self-stigma (lack of)**

1. If I had a mental disorder, I would feel worthless like I had failed my family (33) (SD)
2. I would feel a failure if I had a mental disorder (25) (SD)
3. If I had a mental disorder I would avoid socialising (23) (SD)
4. If I had a mental disorder I would feel ashamed (10 (SD)
