# Supplementary material for: Development and psychometric properties of the Knowledge and Attitudes to Mental Health Scales (KAMHS): a psychometric measure of mental health literacy in children and adolescents
Source: BMC Pediatr. 2021 Nov 13;21:508. doi: 10.1186/s12887-021-02964-x (PMC8590271; doi:10.1186/s12887-021-02964-x)
Supplement: Supplementary file 2 — Additional file 2. Knowledge and Attitudes to Mental Health Scales (Version 3). [file 12887_2021_2964_MOESM2_ESM.docx]

Additional file 2. Knowledge and Attitudes to Mental Health Scales (Version 3).

**For each of the following statements please select which answer (Strongly Agree, Agree, Don’t know, Disagree, or Strongly Disagree) you think is best by making an X in the appropriate box. If you are sure you don’t know the answer then you can put an X in the Don’t Know box.**

Items (item number) Strongly Agree Don’t Disagree Strongly

Agree Know Disagree

**Understanding mental disorders and their treatments**

1. An Anxiety Disorder happens when a person’s brain detects the presence of danger- such as a dog barking (r) (3)
2. Most mental disorders start before the age of 18 (7)
3. Obsessions are thoughts that occur often that the person wants to get rid of but can’t (12)
4. If you are worried about something, then you probably have Generalised Anxiety Disorder (r) (13)
5. Medicines should never be used to treat a mental disorder (r) (15)
6. People with Schizophrenia have a split personality (r) (18)
7. Attention Deficit Hyperactivity Disorder (ADHD) is caused by watching too much TV or eating too much sugar (r) (20)
8. Vitamins and yoga are effective treatments for most mental disorders (r) (26)
9. Emotions are controlled by your heart (r) (28)
10. Severe and repeated stress can affect the brain (29)
11. People with Bipolar Disorder have periods of clinical depression and periods of Mania (31)
12. Schizophrenia can be treated with medication and psychological therapy (32)

**Public Stigma- lack of**

1. If my friend had a mental disorder I would avoid them (r) (2)
2. I would not like to be in the same classroom as someone with a mental disorder (r) (16)
3. I would feel comfortable sitting next to a person with a mental disorder (17)
4. I wouldn’t want to marry or date a person with a mental disorder (r) (27)
5. I would be happy for a person with a mental disorder to come to my house (36)
6. Mental disorders are caused by people being wicked or bad (r) (38)

**Social Desirability**

1. I sometimes think bad thoughts about people (4) (r)
2. I have never dropped rubbish (9)
3. I always keep my promises (14)
4. I always admit when I am wrong (19)
5. I am always honest (22)
6. I always do what my parents/carers asks first time (30)
7. I have said unkind things about a person (41) (r)
8. I always wash my hands before every meal (48)

**Understanding how to optimise and maintain good mental health**

1. The same things that help our physical health also help our mental health (34)
2. Sometimes things that stress you should be faced head-on (35)
3. Healthy eating helps you maintain good mental health (37)
4. A good night’s sleep is good for your mental health (39)
5. Regular exercise has no effect on your mental health (r) (44)
6. Talking about your feelings can help with mental health problems (50)

**Lack of- Avoidant Coping**

1. Drinking alcohol never helps when you are stressed (8)
2. It’s often best to ignore problems and hope they go away (r) (6)
3. Taking illegal drugs can never help when you are stressed by something (21)
4. The best way to cope with problems is not to think about them (r) (40)
5. I do my best not to think about my problems (r) (47)

**Self-stigma- lack of**

1. If I had a mental disorder I would not feel ashamed (10)
2. If I had a mental disorder I would not avoid socialising (23)
3. I would feel a failure if I had a mental disorder (r) (25)
4. If I had a mental disorder, I would feel worthless like I had failed my family (r) (33)
5. I would feel weak if I had a mental disorder (r) (43)
6. If I had a mental disorder, I would feel I’d let everyone down (r) (49)

**Help-seeking behaviours**

1. I wouldn’t tell anyone if I had a mental health problem in case they made fun of me (r) (5)
2. I am confident that I could ask for help if I had a mental health problem (1)
3. For me, it would be easy to ask for help for a mental health problem (11)
4. If I had a mental health problem I would try to hide it from everyone (r) (24)
5. If I had a mental health problem, I would be happy to tell my teacher or school counsellor (42)
6. It’s best not to tell anyone about your mental health problems (r) (45)
7. If I had a mental health problem, I would not tell friends and family (r) (46)

*(r)= reversed scored items.

-For a full copy of the KAMHS questionnaire, please contact the authors directly.
